# Functionalized Linear Conjugated Polymer/TiO_2_ Heterojunctions for Significantly Enhancing Photocatalytic H_2_ Evolution

**DOI:** 10.3390/molecules29051103

**Published:** 2024-02-29

**Authors:** Hao Gong, Yuqin Xing, Jinhua Li, Shiyong Liu

**Affiliations:** Jiangxi Provincial Key Laboratory of Functional Molecular Materials Chemistry, School of Chemistry and Chemical Engineering, Jiangxi University of Science and Technology, Ganzhou 341000, China; 6720210821@mail.jxust.edu.cn (H.G.); xing_yu_qin2@sina.com (Y.X.)

**Keywords:** organic-inorganic heterojunctions, direct C–H arylation polymerization, photocatalysis, linear donor–acceptor conjugated polymers, visible-light-driven hydrogen evolution

## Abstract

Conjugated polymers (CPs) have attracted much attention in recent years due to their structural abundance and tunable energy bands. Compared with CP-based materials, the inorganic semiconductor TiO_2_ has the advantages of low cost, non-toxicity and high photocatalytic hydrogen production (PHP) performance. However, studies on polymeric-inorganic heterojunctions, composed of D-A type CPs and TiO_2_, for boosting the PHP efficiency are still rare. Herein, an elucidation that the photocatalytic hydrogen evolution activity can actually be improved by forming polymeric-inorganic heterojunctions **TFl@TiO_2_**, **TS@TiO_2_** and **TSO_2_@TiO_2_**, facilely synthesized through efficient in situ direct C–H arylation polymerization, is given. The compatible energy levels between virgin TiO_2_ and polymeric semiconductors enable the resulting functionalized CP@TiO_2_ heterojunctions to exhibit a considerable photocatalytic hydrogen evolution rate (HER). Especially, the HER of **TSO_2_@TiO_2_** heterojunction reaches up to 11,220 μmol g^−1^ h^−1^, approximately 5.47 and 1260 times higher than that of pristine **TSO_2_** and TiO_2_ photocatalysts. The intrinsic merits of a donor-acceptor conjugated polymer and the interfacial interaction between CP and TiO_2_ account for the excellent PHP activity, facilitating the separation of photo-generated excitons. Considering the outstanding PHP behavior, our work discloses that the coupling of inorganic semiconductors and suitable D-A conjugated CPs would play significant roles in the photocatalysis community.

## 1. Introduction

The efficient use of solar energy by conserving it in the form of chemical bonds is considered to be one of the most effective ways to build sustainable society. Hydrogen energy, featured by high energy density, high calorific value and zero pollution, has been widely regarded as a clean secondary energy [1,2,3,4]. Solar-driven hydrogen evolution has been developed as a promising means for obtaining renewable energy; meanwhile, the seek for highly active photocatalysts that are capable of efficiently absorbing sunlight to drive the hydrogen evolution reaction (HER) has aroused tremendous interest from scientists. From this perspective, the first discovery of titanium dioxide (TiO_2_) as an inorganic semiconductor photocatalyst, dating back to 1972, reported by Fujishima and Honda [5], laid the foundations and brings infinite opportunities in the field of photocatalysis. Benefiting from the advantages of low cost, non-toxicity, high stability, n-type semiconducting properties, and strong oxidizing ability under light irradiation, TiO_2_ has been considered as the most representative semiconductor photocatalyst and has been widely used in molecular photocatalysis [5] and photocatalyzed hydrogen production (PHP) [6,7,8]. However, the intrinsic narrow photo-response range of TiO_2_ severely limits the light-harvesting ability and causes the rapid recombination of photogenerated electron-hole (e^−^-h^+^) pairs, which has greatly hindered the development of titanium dioxide-based photovoltaic conversion technologies. In order to overcome the abovementioned drawbacks, organic semiconductors have gradually emerged as promising candidates for photocatalysis [9,10,11,12]. Among these, conjugated polymers (CPs), including the g-C_3_N_4_ derivatives [13,14,15], conjugated microporous polymers (CMP) [16,17,18,19,20,21], frameworks of covalent triazines (CTFs) [22,23,24], covalent organic frameworks (COF) [25,26,27,28], and linear conjugated polymers (LCPs) [29,30,31,32], are showcasing the enormous potentials for PHP.

The broad designability of CPs at the molecular-level allows the regulation of structural and electronic properties, which results in narrower bandgap (*E*_g_) and wider absorption range compared to TiO_2_ [33,34]. However, the CP-based photocatalysts still suffer from lower charge carrier mobility because of their shorter exciton diffusion length. In order to settle shortcomings of the single-component photocatalysts, the reasonable exploitation of heterojunctions between CPs and TiO_2_ is desired to be explored, wherein the formed CP@TiO_2_ heterojunctions might take the advantages of CPs and TiO_2_ [35]. As a result, an extended visible-light responsive and the enhanced photoexcited e^−^-h^+^ pairs separation would be expected for the CP@TiO_2_ heterojunctions, thus accelerating the migration of photogenerated charge carriers between CPs and TiO_2_. Such CP@TiO_2_ heterojunctions, in which CPs are mainly g-C_3_N_4_, CMP and COF structures [36,37,38,39,40,41,42,43,44,45,46,47], have showed great potentials in PHP fields.

Donor-acceptor (D-A) conjugated polymers with alternating structures have emerged as promising photocatalysts for hydrogen generation [48,49,50], owing to the intramolecular pull-push effect of D-A structure that would promote electron (e^−^)/hole (h^+^) separation in the presence of light irradiation [51,52,53]. To our knowledge, organic–inorganic heterojunctions composed of D-A type CPs and TiO_2_ have rarely been investigated to date. For example, Chen and Xiang et al. reported that CMP@TiO_2_ heterojunction with D-A motif, in which 1,3,5-triethynylbenzene and thiadiazole derivatives act as D and A unit, respectively, exhibited dramatically increased PHP performance with Pt-cocatalyst [46]. Lately, the same group designed a series of linear conjugated poly(benzothiadiazole) and incorporated onto the surface of TiO_2_ in a similar in situ polycondensation strategy, the resulting heterojunctions consisting of electron-donor and electron-acceptor units embodied superior PHP activity in the absence of a Pt-cocatalyst [54]. In 2019, Xiang and Huang et al. introduced benzene (D) and 2-fluorobenzene(A) to prepare a library of functionalized D-A type heterojunctions in the presence of TiO_2_; the results showed that the HER of functionalized 4% BFBA-TiO_2_ was up to 228.2 μmol h^−1^ [55]. Therefore, it is still challenge to build **CP@TiO_2_** heterojunctions involving D-A architecture whilst little progress has been made. Recently, Wang and Zhang et al. described the fabrication of homopolymer poly (dibenzothiophene-S, S-dioxide) (**PDBTSO**)/TiO_2_ composite composed of A-A structure, exhibiting considerable HER [47]. These meaningful achievements inspired us to explore whether the strategy of introduction D-A conjugated polymers to form TiO_2_-based heterojunction is applicable for improving the PHP performance or not.

Given our consideration in this work, LCPs **TFl**, **TS** and **TSO_2_** were employed as the polymeric “partner” to couple with pristine TiO_2_, and the resulting **CP@TiO_2_** heterojunctions **TFl@TiO_2_**, **TS@TiO_2_** and **TSO_2_@TiO_2_** were prepared with in situ direct C–H arylation polymerization processes. The structural information, opt-electronic and the photocatalytic H_2_ production were investigated. Benefiting from the D-A merits, **TSO_2_@TiO_2_** heterojunction exhibited broader light responsiveness and more efficient photo-excited charge separation and transfer. HER of **TSO_2_@TiO_2_** heterojunction reaches 11,220 μmol h^−1^ g^−1^ under full arc light irradiation, which is 5.47 and 1260 times higher than that of pure polymeric **TSO_2_** and TiO_2_, respectively. Our findings offer an alternative way to fabricate a polymeric heterojunction photocatalyst consisting of a D-A building block for boosting PHP performance.

## 2. Results and Discussion

### 2.1. Synthesis and Characterization of the CPs and CP@TiO_2_ Heterojunctions

**Scheme 1:**
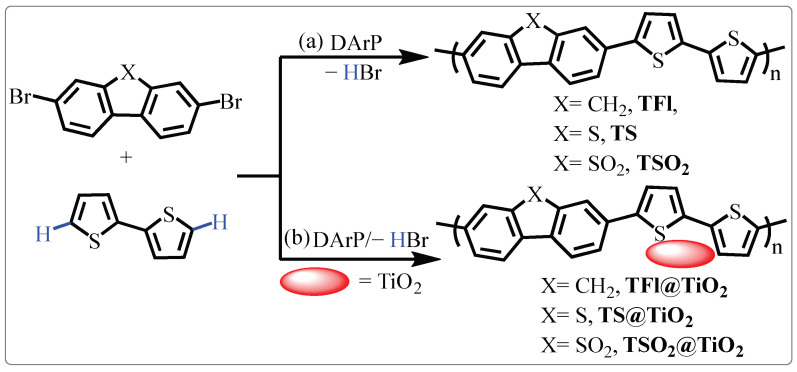
(**a**) Synthesis of LCPs **TFl**, **TS** and **TSO_2_** by DArP polymerization; (**b**) in situ preparation of **TFl@TiO_2_**, **TS@TiO_2_** and **TSO_2_@TiO_2_** heterojunctions.

On the basis of our previous well-developed Pd-catalyzed direct C–H arylation polymerization strategy (DArP) [32,56,57], three pristine LCPs denoted as **TFl**, **TS** and **TSO_2_** showed in Scheme 1a were prepared by treatment of corresponding dibromo-precursors, 2,2-biothiophene via DArP methods, as detailed in the supporting information. Accordingly, the **CP@TiO_2_** heterojunctions (**TFl@TiO_2_**, **TS@TiO_2_** and **TSO_2_@TiO_2_**, Scheme 1b) were fabricated via in situ DArP between appropriate dibromides, 2,2-biothiophene in the presence of TiO_2_. Taking **TSO_2_@TiO_2_** as an example, anhydrous toluene (8 mL) was added to a Schlenk tube containing 3,7-dibromobenzothiophene-S, S-dioxide (40.0 mg, 1 eq), 2,2-biothiophene (19.7 mg, 1 eq), Cs_2_CO_3_ (77.1 mg, 2 eq), PivOH (3.6 mg, 30 mol%), P(*o*-MeOPh)_3_ (2.5 mg, 6 mol%), Pd_2_(dba)_3_ (3.2 mg, 3 mol%) and TiO_2_ (81.0 mg). Then, the resulting mixture was stirred in an oil bath at 100 °C under an argon atmosphere for 48 h. After the reaction was completed, the desired **TSO_2_@TiO_2_** heterojunction was obtained by filtration, followed by continuous washing with dichloromethane-methanol-water and finally drying at 80 °C under vacuum conditions for 24 h.

**Figure 1:**
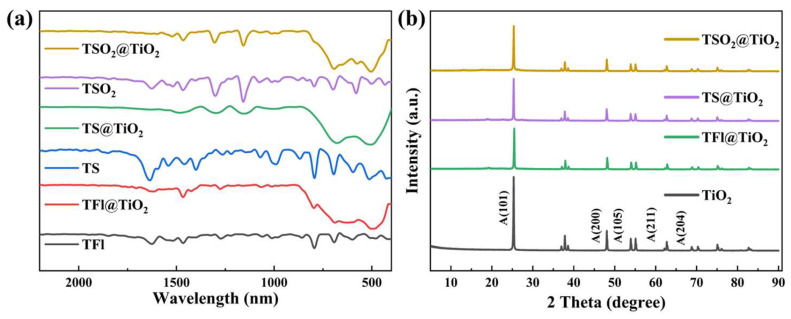
(**a**): FT-IR spectra and (**b**): XRD patterns of CPs (**TFl**, **TS**, **TSO_2_**) and CP/TiO_2_ heterojunctions (**TFl@TiO_2_**, **TS@TiO_2_**, **TSO_2_@TiO_2_**).

The structures of the as-prepared LCPs and CP@TiO_2_ heterojunctions were initially determined by Fourier transform infrared (FT-IR) spectra. As shown in Figure 1a, the broad peaks falling in the range of 500–800 cm^−1^ observed for **TFl@TiO_2_**, **TS@TiO_2_** and **TSO_2_@TiO_2_** are assigned for the Ti–O–Ti stretching vibrations [37,39], which firmly provides evidence for the successful heterojunction interactions between the as-prepared LCPs and TiO_2_. The characteristic signals located at ~1630 cm^−1^, attributing to the stretching vibration of aromatic rings, can be found in the LCPs and CP@TiO_2_ heterojunctions. The characteristic signals displayed at ~1466 cm^−1^ and ~796 cm^−1^ are attributable to the thiophene unit indicate that the bithiophene building block has been introduced into six samples. Additionally, the typical peaks appeared at ~1301 cm^−1^ and ~1157 cm^−1^, belonging to characteristic peaks that for the sulfonyl group (O=S=O), are found in both **TSO_2_** and **TSO_2_@TiO_2_**, respectively.

The formation of CP@TiO_2_ heterojunctions was also determined by powder X-ray diffraction (XRD) patterns. As shown in Figure 1b, the pristine TiO_2_ exhibits representative high and narrow diffraction peaks located at 2θ = 25.3°, 37.8°, 48.0°, 53.9°, 55.1° and 62.7°, corresponding to the (101), (004), (200), (105), (211) and (204) planes of TiO_2_, respectively, which are consistent with the tetragonal anatase TiO_2_ standard card (JCPDS No. 21-1272). In contrast, the **TFl@TiO_2_**, **TS@TiO_2_** and **TSO_2_@TiO_2_** heterojunctions show identical XRD patterns to those of pure TiO_2_, which reveals that the in situ polymerization has a negligible impact on the crystallinity of TiO_2_.

**Figure 2:**
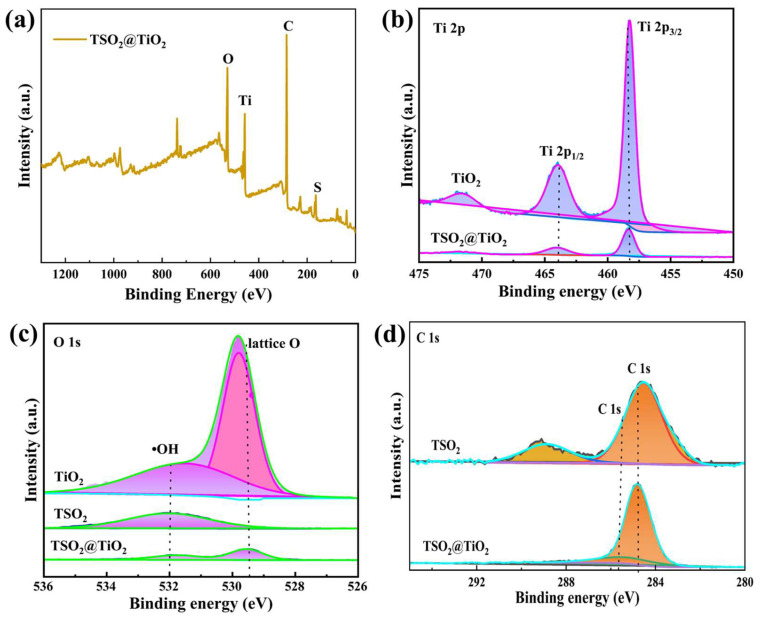
XPS spectra of **TSO_2_@TiO_2_**. (**a**) Survey spectrum, (**b**) Ti 2p, (**c**) O 1s, (**d**) C 1s.

X-ray photoelectron spectroscopy (XPS) measurement was performed to analyze the elemental composition and chemical state of pristine **TiO_2_** and the obtained **TSO_2_** and **TSO_2_@TiO_2_** (Figure 2 and Figure S1). The XPS survey spectrum illustrated in Figure 2a elucidated the existence of Ti, O, C, and S elements in the chemical composition of **TSO_2_@TiO_2_**, suggesting the successful construction of the expected heterojunction. Meanwhile, the corresponding high resolution XPS (HR-XPS) spectra of Ti 2p shown in Figure 2b indicate two similar peaks, which are ascribed to Ti 2p_1/2_ and Ti 2p_3/2_, respectively, for pure **TSO_2_** and **TSO_2_@TiO_2_**. Simultaneously, the HR-XPS spectra of O 1s demonstrates the characteristic peaks for OH and Ti-O-Ti on the surface of TiO_2_ with a slight energy shift for **TSO_2_@TiO_2_** compared to those of **TSO_2_** and TiO_2_ (Figure 2c). Similarly, the HR-XPS C 1s spectra of **TSO_2_@TiO_2_** shows a slightly up-shifted binding energy compared with the polymeric **TSO_2_** (Figure 2d). The change in binding energy indicates the involvement of an interfacial interaction between the **TSO_2_** and TiO_2_, which could accelerate the charge transfer.

**Figure 3:**
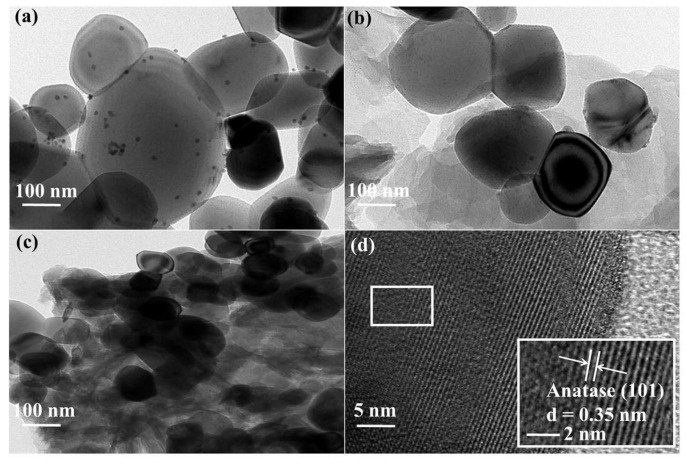
TEM images of (**a**) **TFl@TiO_2_**, (**b**) **TS@TiO_2_** and (**c**,**d**) **TSO_2_@TiO_2_**.

To further investigate the heterojunction morphology of CP@TiO_2_, transmission electron microscopy (TEM) and EDS measurements were carried out next (Figure 3, Figures S2 and S3). Typically, pure TiO_2_ is in the shape of microspheres; however, when composites are formed by coupling with the conjugated polymers, small particles and many folds are observed on the surface (Figure 3a–c). In addition, a high-resolution TEM was carried out on **TSO_2_@TiO_2_** and it was evident that the lattice striations were anatase (101) with a crystalline surface spacing of 0.35 nm (Figure 3d). The EDS elemental analysis of the **TSO_2_@TiO_2_** composite shows the existence of Ti, S, C and O atoms (Figures S2 and S3). Therefore, both high-resolution TEM and EDS clearly prove the successful formation of the **TSO_2_@TiO_2_** heterojunction.

**Figure 4:**
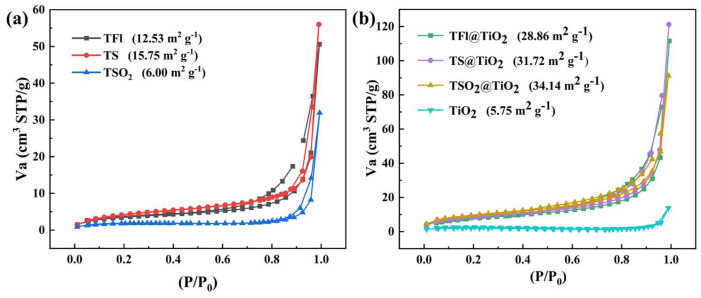
N_2_ adsorption-desorption isothermal profiles of (**a**) **TFl**, **TS** and **TSO_2_**, and (**b**) **TFl@TiO_2_**, **TS@TiO_2_**, **TSO_2_@TiO_2_**, and **TiO_2_**.

The specific Brunauer–Emmett–Teller (BET) surface area of LCPs, CP@TiO_2_ heterojunctions and TiO_2_ was obtained by nitrogen adsorption–desorption isotherms methods (Figure 4), which indicates a descending order in a sequence of **TSO_2_@TiO_2_** > **TS@TiO_2_** > **TFl@TiO_2_**. The relatively high surface area of **TS@TiO_2_**, about six times than of pristine TiO_2_, is favorable for the adsorption of reactant molecules and provides a wider range of sites for photocatalytic reactions.

### 2.2. Opt-Electronic Properties

**Figure 5:**
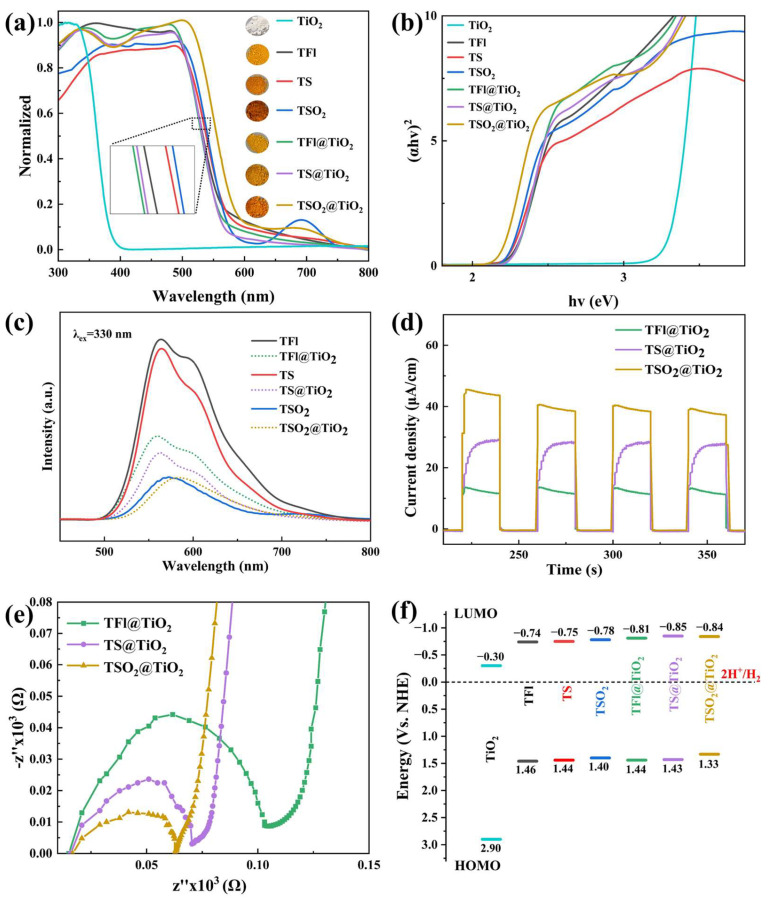
(**a**) UV-vis diffuse reflectance spectra of TiO_2_, **TFl**, **TS**, **TSO_2_** and CP@TiO_2_. (**b**) Kubelka-Munk functions of TiO_2_, **TFl**, **TS**, **TSO_2_** and CP@TiO_2_ with Tauc plots. (**c**) PL spectra. The PL spectra of **TFl**, **TS**, **TSO_2_** and CP@TiO_2_ were measured in the range of 400–850 nm at an excitation wavelength of 330 nm (λ_ex_ = 330 nm); (**d**) transient photocurrent-time (i-t) curve; (**e**) electrical impedance spectra; (**f**) energy band diagrams of TiO_2_, **TFl, TS, TSO_2_** and CP@TiO**_2_** and their proton reduction potentials.

The optical properties of TiO_2_, LCPs and CP@TiO_2_ heterojunctions were first characterized by UV-vis diffuse reflectance spectroscopy (DRS) (Figure 5a), which reveals that both LCPs and CPs@TiO**_2_** heterojunctions have wide and strong light absorption, located in the range of 300 to 600 nm, compared to pristine TiO_2_, enabling them to act as suitable light-harvesting materials. To our delight, **TSO_2_** exhibits a redshift phenomenon compared to those of **TFl** and **TS** among the three LCPs, which is probably due to the fact that **TSO_2_** has an intramolecular donor-acceptor (D-A) interaction induced by the D-A structure. In contrast, **TFl** and **TS** contain D-D structures, thus proving the push–pull electron effect of the D-A interaction is of great significance for broadening the light-absorption. Particularly, the **TSO_2_@TiO_2_** heterojunction possesses the most red-shifted absorption between 300–600 nm, with an extended absorption peak at ~700 nm compared to those of **TFl@TiO_2_** and **TS@TiO_2_**, could be attributable to the tighter connection of the polymer **TSO_2_** to TiO_2_. As can be seen in Figure 5b, the optical band gaps (*E*g) of **TFl**, **TS** and **TSO_2_** are, as calculated by Tauc plots, 2.2, 2.19 and 2.18 eV, respectively. In addition, the *E*g values of TiO_2_ and the CP@TiO_2_ heterojunctions are 3.2 (TiO_2_), 2.25 (**TFl@TiO_2_**), 2.28 (**TS@TiO_2_**) and 2.17 eV (**TSO_2_@TiO_2_**), among which the narrowest *E*g of **TSO_2_@TiO_2_** is consistent with the wide light absorption property.

To evaluate the abilities of photogenerated carrier migration and separation for the obtained LCPs and CP@TiO_2_ heterojunctions, we then carried out the steady-state PL (Figure 5c), TPR (Figure 5d) and EIS (Figure 5e) measurements. The PL intensities for D-A type **TSO_2_** and **TSO_2_@TiO_2_** heterojunction are comparative and relatively lower than those of the remaining four samples, indicating an immense tendency for photo-to-current conversion, thereby resulting in superior separation of the e^−^/h^+^ pairs. In addition, the PL intensities of **TFl@TiO_2_** and **TS@TiO_2_** heterojunctions are significantly lower than the behaviors of their parent conjugated polymers, which are in accordance with the DRS results. Furthermore, the TPR curves showcase that the instantaneous photocurrent-time response of three heterojunctions is in a sequence of **TSO_2_@TiO_2_** > **TS@TiO_2_** > **TFl@TiO_2_**, implying that more light-induced excitons can be generated for the D-A motif containing **TSO_2_@TiO_2_** heterojunction [58,59]. Consistent with the PL and TPR results, the EIS experiments demonstrate that the order of the Nyquist circle radius is **TSO_2_@TiO_2_** < **TS@TiO_2_** < **TFl@TiO_2_**. Therefore, the smallest arc radius of the Nyquist plot for **TSO_2_@TiO_2_** means it has lower interfacial resistance and excellent charge mobility characters [60,61,62].

In combination with cyclic voltammetry (CV) (Figure S4) curves and the equation E_HOMO_ = E_LUMO_ − *E*g, the HOMO and LUMO levels of LCPs and CP@TiO_2_ heterojunctions are estimated. As shown in Figure 5f, the HOMO/LUMO levels of LCPs are 1.46/−0.74 (**TFl**), 1.44/−0.75 (**TS**) and 1.40/−0.78 eV (**TSO_2_**), while their corresponding TiO_2_-based heterojunctions are 1.44/−0.81 (**TFl@TiO_2_**), 1.43/−0.85 (**TS@TiO_2_**) and 1.33/−0.84 eV (**TSO_2_@TiO_2_**), respectively. In contrast, the HOMO/LUMO levels of pristine TiO_2_ are 2.90/−0.30 eV. Consequently, the **TSO_2_@TiO_2_** heterojunction exhibits the narrowest *E*g with a value of 2.17 eV, agreeing with the *E*g calculated using Tauc plots, which provides many opportunities for the **TSO_2_@TiO_2_** heterojunction to achieve the reductive transformation of H^+^ to H_2_. Overall, the opt-electronic properties suggest that **TSO_2_@TiO_2_** heterojunction can serve as a potential efficient photocatalyst for water splitting.

### 2.3. Photocatalytic Hydrogen Production Performances

**Figure 6:**
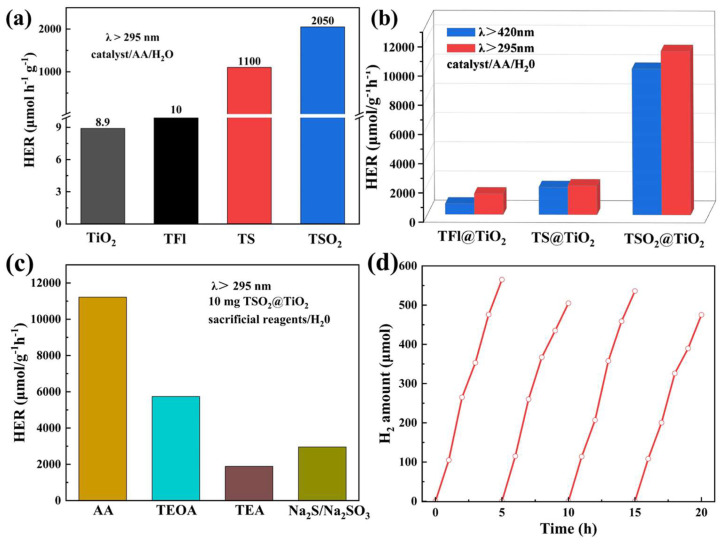
(**a**) Hydrogen production data of **TiO_2_**, **TFl**, **TS** and **TSO_2_** under UV-vis light; (**b**) Hydrogen production data for **TFl@TiO_2_**, **TS@TiO_2_** and **TSO_2_@TiO_2_** under visible light and UV-vis light; (**c**) HER of **TSO_2_@TiO_2_** in different sacrificial agents; (**d**) **TSO_2_@TiO_2_** photocatalytic hydrogen production cycle experiments.

Based on the opt-electronic properties of LCPs and **CP@TiO_2_** heterojunctions, the PHP performances were spontaneously tested under visible or full-arc light irradiation by utilizing the relevant photocatalysts (10 mg) dispersed in N-methyl pyrrolidone (NMP)/H_2_O (30 mL) aqueous solutions, together with the ascorbic acid (AA) as a sacrificial electron donor (SED) [20,32]. As shown in Figure 5a, apparent differences are observed for the normalized hydrogen evolution rates (HERs) of three LCPs under full-arc light irradiation, among which the polymeric **TSO_2_** photocatalyst exhibits the highest PHP activity with HER of 2050 μmol/g^−1^ h^−1^ due to the introduction of the D-A structure. However, the raw material TiO_2_ only shows weak PHP capacity (8.9 μmol/g^−1^ h^−1^). Impressively, enormous improvements in HERs are found when TiO_2_ is coupled with LCPs for **CP@TiO_2_** heterojunctions compared to those of TiO_2_ and their parent LCPs (Figure 6b). Specifically, the HERs in the full-arc band are 1430 (**TFl@TiO_2_**), 2000 (**TS@TiO_2_**) and 11,220 μmol/g^−1^ h^−1^ (**TSO_2_@TiO_2_**), respectively, which indicates that the formation of heterojunction would improve the performance of polymeric photocatalysts because of the charge transfer between the polymer and TiO_2_. In comparison, we also test the PHP activities of **CP@TiO_2_** heterojunctions under the irradiation of visible light (λ > 420 nm) for their intense absorption in this range. As displayed in Figure 6b, the HERs of **TFl@TiO_2_**, **TS@TiO_2_** and **TSO_2_@TiO_2_** are still far exceeded by the corresponding LCPs and TiO_2_, albeit with inferior performance compared with the full-arc band irradiation, proving that the combination of conjugated polymer and TiO_2_ can significantly extend the visible light response. Notably, the HERs of **TSO_2_@TiO_2_** heterojunction are 1260 and 5.47 times higher than those of pristine TiO_2_ and the linear polymeric photocatalyst **TSO_2_**, respectively, which suggests that building D-A architecture-type polymeric and forming organic polymer@TiO_2_ heterojunctions is an effective strategy for improving the performance of PHP for the reason that the interaction between the D-A motif and TiO_2_ could synergistically facilitate exciton diffusion and enhance charge separation for proton reduction.

**Figure 7:**
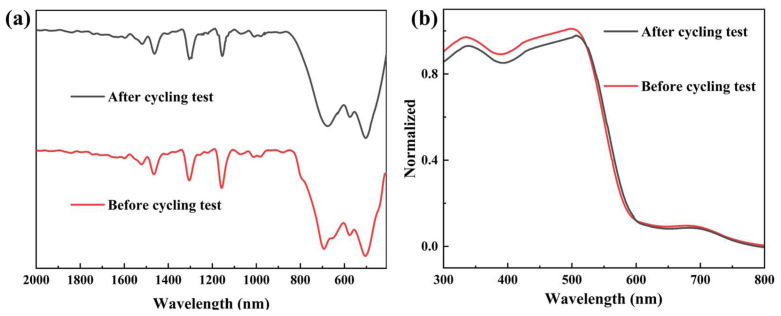
(**a**) FT-IR and (**b**) UV-vis DRS spectra of **TSO_2_@TiO_2_** before and after cycling tests.

To explore the effect of different SEDs on the PHP activities, as illustrated in Figure 6c, TEOA, TEA and Na_2_S/Na_2_SO_3_ were used instead of AA. As a result, a better performance was achieved when AA was employed as the SED. With the optimized condition in hand, the cycling experiments were studied to assess the long-term stability of **TSO_2_@TiO_2_** heterojunction toward the PHP reaction. As depicted in Figure 6d, steady photocatalytic dihydrogen gas generation could be observed through 20 h cycling test with four successive cycles, implying the photochemical stability of the **TSO_2_@TiO_2_** heterojunction. Meanwhile, the **TSO_2_@TiO_2_** was recovered for structural investigation, and no discernible differences were observed from both the UV-vis and FT-IR spectra compared to the as-prepared one (Figure 7a,b), demonstrating the long-term photo-stability of the **TSO_2_@TiO_2_** during the PHP process.

**Figure 8:**
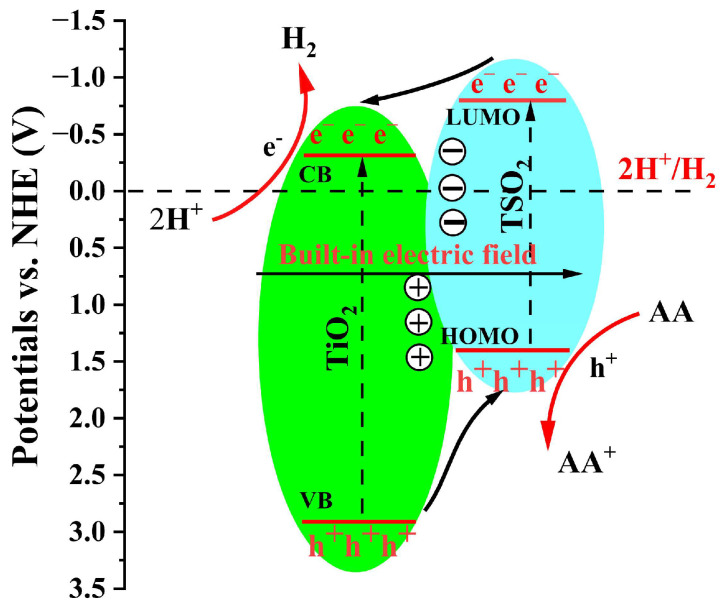
**TSO_2_@TiO_2_** mechanism diagram.

According to the above experimental results, the plausible mechanism for the increased photocatalytic H_2_ evolution of **TSO_2_@TiO_2_** heterojunction is proposed in Figure 8. Since the pure polymeric D-A type **TSO_2_** exhibits more negative LUMO value and less positive value than those of pristine TiO_2_ (Figure 5f), **TSO_2_** rather than TiO_2_ is photo-activated under the illumination of full-arc or visible light (λ > 420 nm). As a result, hole-electron pairs were generated from the **TSO_2_** phase. Meanwhile, some of the photo-generated electrons were inclined to jump from the LUMO energy level of **TSO_2_** to the conduction band of TiO_2_, driven by the built-in electric field, via the interfacial heterojunction, thus subsequently reacting with the protons derived from water to produce H_2_. Besides, the photo-generated holes can easily transfer from the valence band of TiO_2_ to the HOMO energy level of **TSO_2_**, which in turn contributes to oxidizing the AA to AA^+^. Therefore, the effective photo-excited e^−^-h^+^ separation of **TSO_2_@TiO_2_** heterojunction results in enhanced photocatalytic H_2_ generation.

## 3. Materials and Methods

### 3.1. Materials and Methods

All of the starting materials and reagents were purchased from commercial suppliers and used directly without further purification. Anhydrous toluene was pretreated with calcium hydride (CaH_2_) and freshly distilled.

FT-IR spectra were collected on a FT-IR spectrometer (Bruker ALPHA, Billerica, MA, USA) and the KBr was mixed with the sample for sample preparation. The morphology of heterojunction photocatalysts was obtained by scanning electron microscopy (SEM, MLA650F, Hillsboro, OR, USA) and transmission electron microscopy (TEM, FEI Tecnai G2 F20, Thermo Fisher Scientific, Waltham, MA, USA). We recorded polymer photoluminescence (PL) conductivity by employing a HORIBA FL-1000 fluorescence spectrometer for solid powders. EDS uses TESCAN MIRA LMS + Quantax 200 X Flash 6|60. X-ray diffraction (XRD) was measured by the Thermo Fisher NexsaI instrument. X-ray photoelectron spectroscopy (XPS) was measured by the Thermo Fisher NexsaI instrument. The solid UV-visible absorption spectra of the synthesized polymers were detected by a UV-2600 spectrophotometer using BaSO_4_ as a substrate reference. The water contact angle was measured using a JCY type contact angle measuring instrument. Transient photo-response tests were performed using a three-electrode configuration electrochemical workstation (CHI650E/700E, Huachen Co., Ltd., Shanghai, China) to measure transient photocurrents using a Pt electrode as the auxiliary electrode and an Ag/AgCl electrode containing a saturated KCl solution as the reference electrode. The polymer was ultrasonically dispersed with ethanol to form a suspension, which was then dripped onto ITO conductive glass to form a sample with an effective area of 0.6 cm × 0.6 cm, and tested in a 0.1 M aqueous sodium sulfate solution as the electrolyte. Cyclic voltammetry tests were carried out with cyclic voltammetry using an electrochemical workstation (CHI650E/700E, Huachen Co., Ltd., Shanghai, China) with a three-electrode system using a glassy carbon electrode as the working electrode, an Ag/AgCl electrode as the reference electrode, and a Pt electrode as the auxiliary electrode, and an electrolyte was prepared by dissolving the TBAPF6 in acetonitrile solution, and the electrolyte was then used as an electrophoretic solution according to the equation: E_HOMO_ = −(E^OX^ + 4.8 eV(νs Ag/Ag^+^) − E^OX^_Fc/Fc+_), E_HOMO_ was calculated from the curve. The volume of nitrogen adsorption was recorded over a relative pressure range between 0.01 and 0.99 points in the relative pressure range of 0.05–0.2 were used for the calculation of the surface area according to the Brunauer-Emmet-Teller (BET) theory.

### 3.2. Synthesis of 3,7-Dibromodibenzothiophene-S,S-dioxide [63]

NBS (2.47 g, 13.87 mmol) was added into this solution of dibenzothiophene-S, S-dioxide (1.5 g, 6.94 mmol) in concentrated H_2_SO_4_ (90 mL) in several portions, and the resulting mixture was stirred at 0–5 °C for 24 h. The mixture was carefully poured into ice/water. The off-white solid was filtered off, washed with 20% aqueous sodium hydrogen carbonate, water and dried to an afford white solid. The product was further recrystallized from chloroform to gain a white crystal with a 60% yield. ^1^H-NMR (400 MHz, CDCl_3_) δ 7.93 (s, 2H), 7.78 (d, 2H), 7.65 (d, 2H). ^13^CNMR (400 MHz, CDCl_3_) δ 138.96, 137.25, 129.68, 125.70, 123.02.

### 3.3. Synthesis of **TFl, TS, TSO_2_, TiO_2_, TFl@TiO_2_, TS@TiO_2_ and TSO_2_@TiO_2_**

**TFl**: A mixture of 2,7-dibromofluorene (100.0 mg, 1.0 eq), 2,2-biothiophene (51.3 mg, 1.0 eq), Cs_2_CO_3_ (100.6 mg, 2.0 eq), PivOH (4.7 mg, 30 mol%), P(o-MeOPh)_3_ (2.2 mg, 4 mol%) and Pd_2_(dba)_3_ (2.8 mg, 2 mol%) was added to 5 mL of anhydrous toluene under an argon atmosphere, and the mixture was degassed in three freeze-vacuum-thaw cycles. The reaction was then stirred in an oil bath at 100 °C for 48 h. After the reaction was completed and cooled, the product was washed and filtered using dichloromethane–methane-water in sequence to remove inorganic salts and residual small molecules. The reaction was then dried in a vacuum oven at 80 °C for 24 h, 115.8 mg, yield: 91%.

**TS**: A mixture of 3,7-dibromodibenzothiophene (100.0 mg, 1.0 eq), 2,2-biothiophene (48.6 mg, 1.0 eq), Cs_2_CO_3_ (190.5 mg, 2.0 eq), PivOH (8.9 mg, 30 mol%), P(o-MeOPh)_3_ (2.2 mg, 4 mol%) and Pd_2_(dba)_3_ (2.8 mg, 2 mol%) was added to 5 mL of anhydrous toluene under an argon atmosphere and the mixture was degassed in three freeze-vacuum-thaw cycles. The reaction was then stirred in an oil bath at 100 °C for 48 h. After the reaction was completed and cooled, the product was washed and filtered using dichloromethane-methane-water in sequence to remove inorganic salts and residual small molecules. The reaction was then dried in a vacuum oven at 80 °C for 24 h, 118.3 mg, yield: 95%.

**TSO_2_**: A mixture of 3,7-dibromodibenzothiophene-5,5-dioxide (100.0 mg, 1.0 eq), 2,2-biothiophene (44.4 mg, 1.0 eq), Cs_2_CO_3_ (174.2.5 mg, 2.0 eq), PivOH (8.2 mg, 30 mol%), P(o-MeOPh)_3_ (3.8 mg, 4 mol%) and Pd_2_(dba)_3_ (4.9 mg, 2 mol%) was added to 5 mL of anhydrous toluene under an argon atmosphere, and the mixture was degassed in three freeze-vacuum-thaw cycles. The reaction was then stirred in an oil bath at 100 °C for 48 h. After the reaction was completed and cooled, the product was washed and filtered using dichloromethane–methane-water in sequence to remove inorganic salts and residual small molecules. The reaction was then dried in a vacuum oven at 80 °C for 24 h, 115.7 mg, yield: 94%.

**TFl@TiO_2_:** A mixture of 2,7-dibromofluorene (40.0 mg, 1.0 eq), 2,2-biothiophene (20.5 mg, 1.0 eq), Cs_2_CO_3_ (80.4 mg, 2.0 eq), PivOH (3.8 mg, 30 mol%), P(o-MeOPh)_3_ (2.6 mg, 6 mol%), Pd_2_(dba)_3_ (3.4 mg, 3 mol%) and TiO_2_ (81.0 mg) was added to 8 mL of anhydrous toluene under an argon atmosphere, and the mixture was degassed in three freeze-vacuum-thaw cycles. The reaction was then stirred in an oil bath at 110 °C for 48 h. After the reaction was completed and cooled, the product was washed and filtered using dichloromethane–methane-water in sequence to remove inorganic salts and residual small molecules. The reaction was then dried in a vacuum oven at 80 °C for 24 h, 113.9 mg, yield: 87%.

**TS@TiO_2_:** A mixture of 3,7-dibromodibenzothiophene (40.0 mg, 1.0 eq), 2,2-biothiophene (19.4 mg, 1.0 eq), Cs_2_CO_3_ (76.2 mg, 2.0 eq), PivOH (3.6 mg, 30 mol%), P(o-MeOPh)_3_ (2.4 mg, 6 mol%), Pd_2_(dba)_3_ (3.2 mg, 3 mol%) and TiO_2_ (81.0 mg) was added to 8 mL of anhydrous toluene under an argon atmosphere, and the mixture was degassed in three freeze-vacuum-thaw cycles. The reaction was then stirred in an oil bath at 110 °C for 48 h. After the reaction was completed and cooled, the product was washed and filtered using dichloromethane–methane-water in sequence to remove inorganic salts and residual small molecules. The reaction was then dried in a vacuum oven at 80 °C for 24 h, 119.2 mg, yield: 91%.

**TSO_2_@TiO_2_:** A mixture of 3,7-dibromobenzothiophene-5,5-dioxide (40.0 mg, 1.0 eq), 2,2-biothiophene (19.7 mg, 1.0 eq), Cs_2_CO_3_ (77.1 mg, 2.0 eq), PivOH (3.6 mg, 30 mol%), P(o-MeOPh)_3_ (2.5 mg, 6 mol%), Pd_2_(dba)_3_ (3.2 mg, 3 mol%) and TiO_2_ (81.0 mg) was added to 8 mL of anhydrous toluene under an argon atmosphere, and the mixture was degassed in three freeze-vacuum-thaw cycles. The reaction was then stirred in an oil bath at 110 °C for 48 h. After the reaction was completed and cooled, the product was washed and filtered using dichloromethane–methane-water in sequence to remove inorganic salts and residual small molecules. The reaction was then dried in a vacuum oven at 80 °C for 24 h, 116.5 mg, yield: 89%.

### 3.4. PHP Tests

A gas chromatograph (GC9790, FuLi, Wenzhou, China) was linked with the photocatalytic online analysis system (LabSolar-III AG, Beijing Perfect Light, Beijing, China) for the typical PHP test; it was equipped with the thermal conductive detector (TCD) and used argon as the carrier gas. A mixed aqueous solution containing 30 mL H_2_O and 5 g AA as the sacrificial agent was prepared for the photocatalysts **TFl, TS, TSO_2_, TiO_2_, TFl@TiO_2_, TS@TiO_2_ and TSO_2_@TiO_2_** (10 mg) ultrasonic dispersal. The oil pump was used to remove the dissolved air form the mixture and maintain it in vacuum. To irradiate the reaction vessel, a 300 W Xe lamp (Beijing Perfect Light, PLS-SXE300) under full-arc light irradiation was used. To fix the reaction temperature at 25 °C, a flow of cooling water was used.

## 4. Conclusions

In summary, three LCPs entitled **TFl**, **TS** and **TSO_2_** were facile synthesized via the atom-economic DArP methodology, featuring D-D (for **TFl** and **TS**) and D-A (for **TSO_2_**) architectures, respectively. Additionally, compatible **CP@TiO_2_** heterojunctions **TFl@TiO_2_, TS@TiO_2_** and **TSO_2_@TiO_2_** were successfully constructed through in situ DArP routes in the presence of TiO_2_. The chemical structures and compositions of the expected LCPs and CP@TiO_2_ heterojunctions were characterized by FT-IR, XRD, XPS and TEM. The investigation, including DRS, PL, TPR, EIS and CV measurements, on the opt-electronic properties revealed that the **TSO_2_@TiO_2_** heterojunction can serve as an ideal polymeric photocatalyst candidate due to its increased photo-responsive and separated electron-hole pairs, inducing the intramolecular D-A interaction. Impressively, the PHP tests showed that the desired photocatalyst **TSO_2_@TiO_2_** heterojunction presented a remarkable photocatalytic HER of 11,220 μmol g^−1^ h^−1^ under full arc irradiation, which is 1260 and 5.47 times higher than that of pristine TiO_2_ and **TSO_2_** photocatalysts, respectively. A plausible mechanism involving electron and hole transfer between polymeric **TSO_2_** and TiO_2_ was raised to elucidate the enhancement of PHP performance. Our results not only demonstrate that the D-A conjugated polymer and TiO_2_ heterostructures bring tremendous opportunities for PHP, but also provide a feasible avenue to seek novel photocatalysts.

## Data Availability

Data are contained within the article and Supplementary Materials.

## Supplementary Materials

The following supporting information can be downloaded at: https://www.mdpi.com/article/10.3390/molecules29051103/s1, Scheme S1： Synthesis of 3,7-dibromodibenzothiophene-S, S-dioxide; Figure S1: XPS plots of TiO_2_, **TSO_2_** and **TSO_2_@TiO_2_**; Figure S2: TEM plots of **TSO_2_@TiO_2_**; Figure S3: EDS plots of **TSO_2_@TiO_2_**; Figure S4: CV plots of (a) **TFl** (b) **TS** (c) **TSO_2_** (d) **TFl@TiO_2_** (e) **TS@TiO_2_** (f) **TSO_2_@TiO_2_**.
