# Supplementary material for: Functionalized Linear Conjugated Polymer/TiO2 Heterojunctions for Significantly Enhancing Photocatalytic H2 Evolution
Source: Molecules. 2024 Feb 29;29(5):1103. doi: 10.3390/molecules29051103 (PMC10935027; doi:10.3390/molecules29051103)
Supplement: Supplementary file 1 [file molecules-29-01103-s001.zip › molecules-2882020-supplementary.pdf]

Supporting Information

Functionalized Linear Conjugated Polymer/TiO_2 Heterojunctions for Significantly Enhancing Photocatalytic H_2 Evolution

Hao Gong†, Yu-Qin Xing†, Jinhua Li * and Shi-Yong Liu *

Jiangxi Provincial Key Laboratory of Functional Molecular Materials Chemistry, School of Chemistry and Chemical Engineering, Jiangxi University of Science and Technology, Ganzhou 341000, China; 6720210821@mail.jxust.edu.cn (H. Gong); xing_yu_qin2@sina.com (Y.-Q. Xing)

* Correspondence: lijh@jxust.edu.cn (J. Li); chelsy@jxust.edu.cn or chelsy@zju.edu.cn (S.-Y. Liu)

† These authors contributed equally to this work.

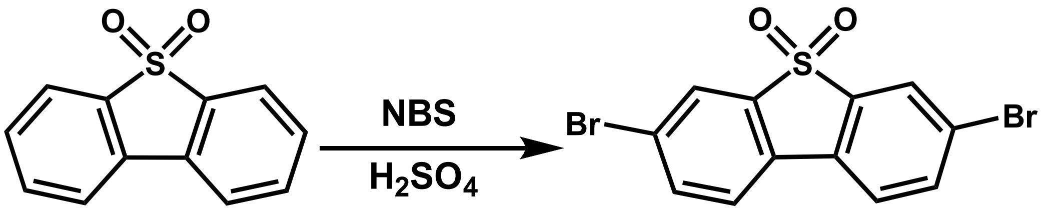

Scheme S1: Synthesis of 3,7-dibromodibenzothiophene-S, S-dioxide

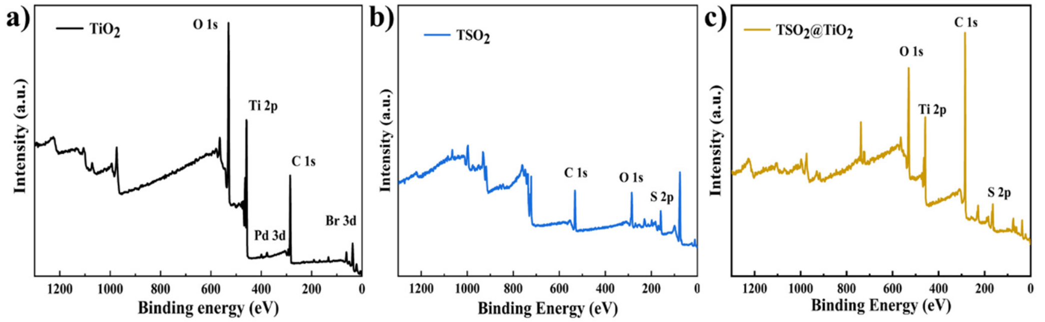

Figure S1: XPS plots of TiO2 **TiO2**, **TSO2** and **TSO2@TiO2**

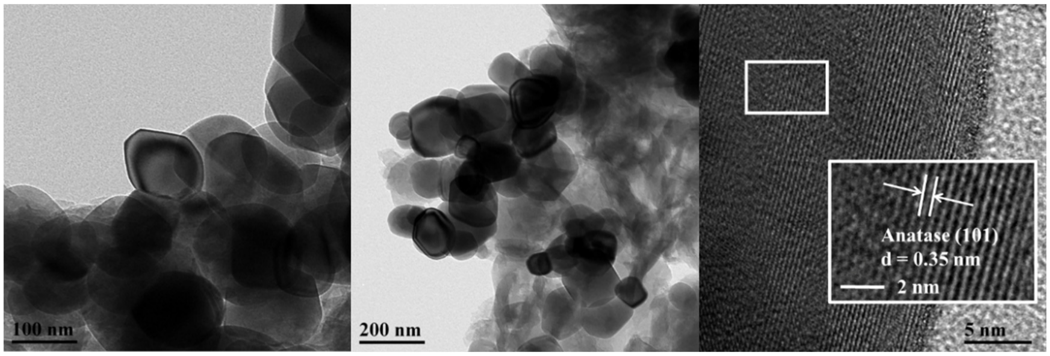

Figure S2: TEM plots of **TSO2@TiO2**

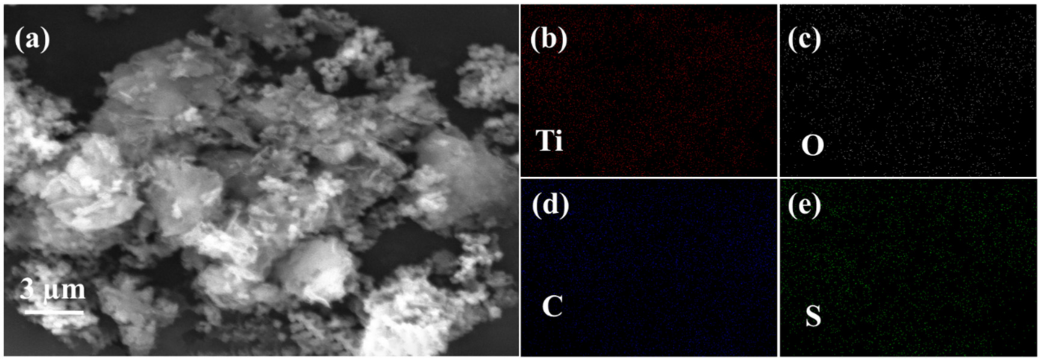

Figure S3: EDS plots of **TSO2@TiO2**

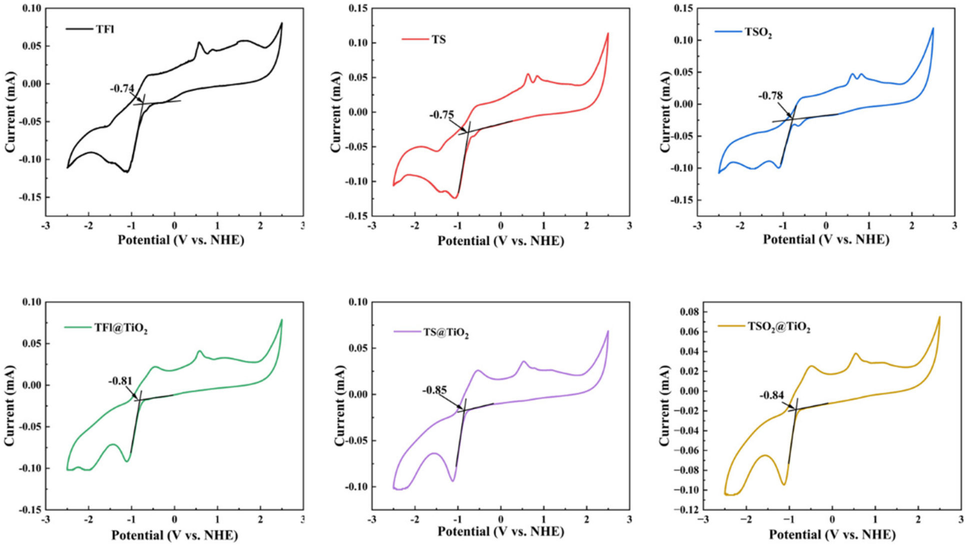

Figure S4: CV plots of (a)**TFl** (b) **TS** (c)**TSO_2** (d)**$TFl@TiO_2$** (e)**$TS@TiO_2$** (f)**$TSO_2@TiO_2$**
